# Associations of Toenail Arsenic, Cadmium, Mercury, Manganese, and Lead with Blood Pressure in the Normative Aging Study

**DOI:** 10.1289/ehp.1002805

**Published:** 2011-08-30

**Authors:** Irina Mordukhovich, Robert O. Wright, Howard Hu, Chitra Amarasiriwardena, Andrea Baccarelli, Augusto Litonjua, David Sparrow, Pantel Vokonas, Joel Schwartz

**Affiliations:** 1 Department of Epidemiology, Gillings School of Global Public Health, University of North Carolina, Chapel Hill, North Carolina, USA; 2 Department of Environmental Health, Harvard School of Public Health, Boston, Massachusetts, USA; 3 Channing Laboratory, Department of Medicine, Brigham and Women’s Hospital, Harvard Medical School, Boston, Massachusetts, USA; 4 Department of Environmental Health Sciences, School of Public Health, University of Michigan, Ann Arbor, Michigan, USA; 5 Department of Environmental and Occupational Health, IRCCS Maggiore Hospital, Mangiagalli and Regina Elena Foundation and Università degli Studi di Milano, Milan, Italy; 6 Veterans Administration Normative Aging Study, Veterans Affairs Boston Healthcare System and the Department of Medicine, Boston University School of Medicine, Boston, Massachusetts, USA

**Keywords:** arsenic, blood pressure, cadmium, epidemiology, lead, manganese, mercury, metals

## Abstract

Background: Arsenic, cadmium, mercury, and lead are associated with cardiovascular disease in epidemiologic research. These associations may be mediated by direct effects of the metals on blood pressure (BP) elevation. Manganese is associated with cardiovascular dysfunction and hypotension in occupational cohorts.

Objectives: We hypothesized that chronic arsenic, cadmium, mercury, and lead exposures elevate BP and that manganese lowers BP.

Methods: We conducted a cross-sectional analysis of associations between toenail metals and BP among older men from the Normative Aging Study (*n* = 639), using linear regression and adjusting for potential confounders.

Results: An interquartile range increase in toenail arsenic was associated with higher systolic BP [0.93 mmHg; 95% confidence interval (CI): 0.25, 1.62] and pulse pressure (0.76 mmHg; 95% CI: 0.22, 1.30). Positive associations between arsenic and BP and negative associations between manganese and BP were strengthened in models adjusted for other toenail metals.

Conclusions: Our findings suggest associations between BP and arsenic and manganese. This may be of public health importance because of prevalence of both metal exposure and cardiovascular disease. Results should be interpreted cautiously given potential limitations of toenails as biomarkers of metal exposure.

Arsenic, cadmium, mercury, and lead are associated with cardiovascular disease in epidemiologic research (Engel et al. 1994; Houston 2007; Navas-Acien et al. 2007). These associations may be mediated by direct effects on blood pressure (BP) elevation. In animals, arsenic, cadmium, mercury, and lead induce hypertension (Navas-Acien et al. 2007; Satarug et al. 2006; Wakita 1987; Yang et al. 2007), and manganese causes hypotension (Jiang and Zheng 2005). All five metals are plausibly linked with BP, based on mechanistic and experimental data (Houston 2007; Jiang and Zheng 2005; Navas-Acien et al. 2007; Valko et al. 2005).

Epidemiologic studies show consistent associations between high arsenic exposure and BP (Chen et al. 1995; Rahman et al. 1999). Few studies have assessed lower-level exposure. Research on cadmium and BP is inconsistent (Staessen et al. 2000; Tellez-Plaza et al. 2008). Lead is a known risk factor for BP elevation (Navas-Acien et al. 2007), but no studies have evaluated toenail lead. High manganese exposure is associated with cardiovascular dysfunction and hypotension in occupational cohorts (Jiang and Zheng 2005). Research at lower exposures is inconsistent (Gonzalez-Muñoz et al. 2010; Taneja and Mandal 2007). Studies examining mercury and BP report mixed results (Johansson et al. 2002; Pedersen et al. 2005; Valera et al. 2009). Data on low-level mercury are sparse.

We hypothesized that chronic arsenic, cadmium, mercury, and lead exposures elevate BP and that manganese lowers BP. We evaluated these hypotheses in a cohort of elderly men from the Greater Boston area.

## Materials and Methods

*Study population.* Subjects are from the ongoing, longitudinal Veterans Administration Normative Aging Study (NAS) (Bell et al. 1972). Participants are males with no known chronic medical conditions at recruitment. They are evaluated at study visits every 3–5 years. This study has been approved by all relevant institutional review boards. All participants gave their written informed consent.

We asked NAS participants to bring toenail clippings to their study visit between January 1999 and January 2009 (*n* = 818 eligible NAS participants). For our analysis, we excluded NAS participants who did not bring toenail clippings (*n* = 165) or were missing information on BP (*n* = 3), race/ethnicity (*n* = 12), education (*n* = 22), alcohol intake (*n* = 11), age (*n* = 1), body mass index (BMI; *n* = 1), smoking history (*n* = 1), pack-years (*n* = 1), or season of clinical visit (*n* = 1). Analyses included 639 men with toenail samples and complete information on BP and relevant covariates on at least one visit.

For each metal, we used data from first visits of the participants with complete information on the specific metal of interest, BP, and relevant covariates in regressions against BP. In contrast, when reporting results for the study population as a whole (i.e., participant characteristics, toenail metal concentrations, and correlations between toenail metals), we used data from the first study visit of the participants with donated toenails (even if some metal concentrations were missing) and complete information on BP and covariates. Many NAS participants had more than one study visit between 1999 and 2009. Sometimes, data were missing on only certain metals at a given visit. Thus, the same participant may contribute data from different visits for, for example, arsenic–BP versus cadmium–BP regressions. Hence, sample sizes sometimes differ across metal–BP regressions even after adjustment for all other toenail metals.

*Physical parameters and medical history.* We asked participants to fast and abstain from smoking overnight prior to their morning visit. Height and weight were measured during physical examinations. Information on smoking and medications was obtained from questionnaires and confirmed by an on-site physician. Alcohol and seafood intakes were determined using a standardized semiquantitative food frequency questionnaire (Willett et al. 1985).

*Toenail samples.* Toenails were collected from all toes, sonicated for 15 min in ~ 10 mL 1% Triton X-100 solution, rinsed with distilled deionized water, dried at 60°C in a drying oven for 24 hr, and weighed. Mercury concentrations were measured by a different assay than the other metals. A sufficient quantity of toenails was not always available for both assays. If weight was > 0.1 g, a portion of the sample was used for mercury analysis, and the rest was acid-digested and analyzed for multi-elements. Samples weighing < 0.1 g were not analyzed for mercury.

Among men otherwise eligible for inclusion in metal–BP regressions, some were excluded because of cadmium concentrations below the detection limit (DL; *n* = 41) and/or samples of insufficient weight to assess metal concentrations (7 for cadmium, 72 for mercury). One man had two otherwise eligible study visits during the period of toenail collection but was excluded from cadmium–BP regressions because of cadmium concentration below the DL in one visit and insufficient sample weight in the other visit. No eligible participants were excluded because of metal concentrations below the DL for arsenic, mercury, manganese, or lead, or because of samples of insufficient weight for arsenic, manganese, or lead. We found no meaningful differences in characteristics between those with sufficient and insufficient sample weight for mercury assays (data not shown).

*Arsenic, cadmium, manganese, lead.* Samples were dissolved in HNO_3_ acid for 24 hr, diluted to 5 mL with deionized water, and analyzed by an inductively coupled plasma-mass spectrometer (Elan 6100; PerkinElmer, Norwalk, CT, USA). Analyses were conducted through external calibration with thalium, indium, and tellurium as internal standards for lead, cadmium, and manganese, and arsenic, respectively.

Quality control (QC) measures included analysis of the initial calibration verification standard (National Institute of Standard and Technology Standard Reference Material 1643e, trace elements in water; Gaithersburg, MD, USA), a 1-ng/mL mixed-element standard solution, continuous calibration standards, and a procedural blank. Certified reference material GBW 07601 was used as the QC sample; we used a large preparation (2 g/L) to monitor daily variation. Results were given as the average of five replicate measurements. Recovery of the analysis of the QC standard by this procedure was 90–110% with 95% precision.

The between-assay coefficient of variation was 3.6% for lead, 3.0% for cadmium, 3.3% for manganese, and 10% for arsenic. The DL for the analytical solution for all four metals was 0.2 × 10^–3^ µg/mL. The DL for the sample depends on sample weight. Sample weight varied from 0.002 g to 0.9 g, and the DL varied from 0.001 µg/g to 0.42 µg/g (mean = 0.02 µg/g).

*Mercury.* We conducted assays with the Direct Mercury Analyzer 80 (Milestone Inc., Monroe, CT, USA). Samples were analyzed using a matrix-matched calibration curve created with different weights of certified reference material GBW 07601 (human hair; Institute of Geophysical and Geochemical Exploration, Langfang, Hebei Province, People’s Republic of China).

QC steps included daily calibration verification of the high and low ends of the calibration curve, a procedural blank, and analysis of National Institute for Environmental Studies certified reference material 13 (human hair; Ibaraki, Japan). Mercury recovery was 90–110%, with > 90% precision.

The DL for the mercury analysis was 0.5 × 10^–3^ µg. The DL for the sample varied according to sample weight. Sample weight varied from 0.01 g to 0.15 g, and the DL varied from 0.003 µg/g to 0.05 µg/g (mean = 0.018 µg/g).

*BP measurements.* Systolic BP (SBP) and fifth-phase diastolic BP (DBP) were measured by a physician at the same visit as toenail collection. BP readings were taken in each arm (to the nearest 2 mmHg) with standard mercury sphygmomanometers with 14-cm cuffs. The means of the arm measurements were used as the SBP and DBP of the participants, and pulse pressure was calculated as SBP – DBP. BP was taken seated, immediately after a seated patient history.

*Statistical methods.* We examined cross-sectional relations between toenail metals and BP from the same study visit using multivariable linear regression.

Antihypertensive medication use is a potential source of bias when examining associations with BP. People taking antihypertensives have controlled and likely artificially low BP. We address this by adding a constant (10 mmHg) to the SBP and DBP of participants using antihypertensives (Cui et al. 2003; Tobin et al. 2005). To test the robustness of our results, we ran a sensitivity analysis examining associations between toenail metals and BP without adding a constant to the SBP and DBP of antihypertensive users. Results were similar when not correcting for medication use (data not shown).

We examined interactions with statins for metals associated with BP in any regression by adding interaction terms between metals and statin use (yes/no) to main effects models and by modeling stratified regressions.

The following potential confounders were identified from the literature and included in all models: age, cigarette smoking (never, current, or former), pack-years, BMI (kilograms per square meter), alcohol intake (two or more vs. fewer than two drinks/day), race/ethnicity, education (years), and season and year of clinical visit. For mercury, we adjusted for dark-meat fish, shellfish, canned tuna, and other fish intake. To evaluate potential confounding by other toenail metals, we included all five metals together in the same regressions, retaining all covariates.

To examine dose–response relationships between arsenic and BP, we refit regression models using the generalized additive model in R (R Project for Statistical Computing, Vienna, Austria). We fit a penalized spline and chose the optimum penalty using generalized cross validation.

We evaluated associations between an interquartile range (IQR) increase in toenail metals and hypertension (defined as antihypertensive medication use or SBP ≥ 140 mmHg or DBP ≥ 90 mmHg at study visit) using logistic regression and adjusting for the aforementioned confounders.

We conducted a sensitivity analysis using inverse probability weighting to adjust for potential selection bias (Hogan and Lancaster 2004). We ran a logistic regression assessing relations between covariates and participation status, retaining variables that were significant at the *p* < 0.10 level (age, BMI, smoking). We obtained predicted probability of participation for each subject, and weighted regression models by inverses of these probabilities. Results were not appreciably altered by inverse probability weighting (data not shown).

Because a number of otherwise eligible participants were excluded from cadmium-BP regressions due to toenail concentrations below the DL, we also conducted a sensitivity analysis in which we assigned participants with cadmium concentrations below the DL a concentration value of the mean sample DL/2 (0.01 µg/g).

Using Student’s *t*-test and chi-square analysis, we compared participants with nonparticipants presenting during the same time period. For this comparison, we used data from the first NAS visit of the nonparticipants between January 1999 and January 2009 (the period of toenail collection). We also calculated medians and IQRs for toenail metal levels after stratifying by potential confounders and effect modifiers. Correlations between toenail metals were evaluated using Spearman correlation. Regression results were similar, although somewhat weaker, when using log-transformed metal concentrations (data not shown).

## Results

*Study population.* At their first study visit with donated toenails and complete information on BP and covariates, participants were mostly former smokers. Mean BMI was 28 kg/m^2^, and 20%, 53%, and 27% of participants had a BMI of < 25, 25–30, and ≥ 30 kg/m^2^, respectively. Participants were younger than nonparticipants (mean ages 72 and 74, respectively, *p* = 0.03) (Table 1). Mean uncorrected SBP and DBP were 133 mmHg and 78 mmHg, respectively, with 71% of participants classified as hypertensive based on medication use or BP at study visit. Sixty percent used antihypertensives; 37% used statins.

**Table 1:** Descriptive statistics by participation status.

Characteristic	Participants *n* = 639	Nonparticipants *n* = 179	*p*-Value
Age (years)		72 ± 6.6		74 ± 7.6		0.03
BMI (kg/m^2^)						0.38
< 25		130 ± 20		33 ± 19		
25–29		338 ± 53		88 ± 49		
≥ 30		171 ± 27		57 ± 32		
Smoking status						0.17
Never smoker		186 (29)		43 (24)		
Former smoker		430 (67)		124 (70)		
Current smoker		23 (3.6)		11 (6.2)		
Smoking (pack-years)		30 ± 28		31 ± 24		0.62
Years of education		15 ± 2.8		14 ± 2.9		0.96
Race/ethnicity						0.85
Non-Hispanic white		624 ± 98		162 ± 97		
Non-Hispanic black		10 ± 1.6		4 ± 2.4		
Hispanic white		4 ± 0.6		1 ± 0.6		
Hispanic black		1 ± 0.2		0		
Alcohol intake (drinks/day)						
< 2		513 ± 80		138 ± 82		0.59
≥ 2		126 ± 20		30 ± 18		
Year of clinical visit		2001 ± 2.1		2001 ± 2.4		0.28
Season of clinical visit						0.10
Spring		164 (26)		31 (17)		
Summer		169 (26)		57 (32)		
Fall		187 (29)		59 (33)		
Winter		119 (19)		31 (17)		
Antihypertensive use		384 (60)		121 (68)		0.06
Statin use		235 (37)		64 (36)		0.84
BP (mmHg)						
SBP		133 ± 17		133 ± 18		0.97
DBP		78 ± 9.3		77 ± 10		0.54
Pulse pressure		55 ± 14		56 ± 16		0.66
Data are given as mean ± SD or *n* (%). Participant data are from first study visit with donated toenails and complete information on BP and covariates. For comparison with eligible NAS subjects who were not included in the metal–BP analyses (i.e., nonparticipants), we used data from first NAS visit of nonparticipants between January 1999 and January 2009 (the period of toenail collection).						

Detailed information on metal concentrations by participant characteristics is provided in Supplemental Material, Table 1 (http://dx.doi.org/10.1289/ehp.1002805). For example, arsenic was higher among men consuming two or more versus fewer than two alcoholic drinks per day (0.09 vs. 0.07 µg/g); among non-Hispanic blacks compared with non-Hispanic whites (0.09 vs. 0.08 µg/g), cadmium was higher among men consuming two or more versus fewer than two alcoholic drinks per day (0.02 vs. 0.01 µg/g) and among men with fewer years of education (< 15 years vs. ≥ 15 years, 0.02 vs. 0.01 µg/g); mercury was higher among men with greater dark-meat fish (0.31 vs. 0.15 µg/g), other fish (0.26 vs. 0.15 µg/g), shellfish (0.27 vs. 0.18 µg/g), and tuna intake (0.25 vs. 0.12 µg/g) (one or more servings per month vs. less than one serving per month for each seafood category). Manganese was lower among statin users (0.24 vs. 0.31 µg/g) and was higher among non-Hispanic whites compared with non-Hispanic blacks (0.28 vs. 0.18 µg/g); lead was higher among men consuming two or more versus fewer than two alcoholic drinks per day (0.38 vs. 0.30 µg/g) and among men with fewer years of education (< 15 years vs. ≥ 15 years, 0.35 vs. 0.26 µg/g). Toenail cadmium levels were similar across categories defined by smoking history or pack-years [Supplemental Material, Table 1 (http://dx.doi.org/10.1289/ehp.1002805)], even when participants with cadmium concentrations below the DL were assigned a concentration value of 0.01 µg/g (mean sample DL/2; data not shown). Correlations between toenail metals are reported in Supplemental Material, Table 2 (http://dx.doi.org/10.1289/ehp.1002805). Briefly, we found statistically significant correlations between arsenic and cadmium (*r* = 0.39), mercury (*r* = 0.09), manganese (*r* = 0.56), and lead (*r* = 0.45), between cadmium and manganese (*r* = 0.49) and lead (*r* = 0.58), and between manganese and lead (*r* = 0.50) (Supplemental Material, Table 2).

**Table 2:** Linear regression models estimating the change in BP parameters associated with an IQR increase in metal levels.*a,b*

Change in BP (mmHg, 95% CI)												
Toenail metal	Median (μg/g)	Range (μg/g)	IQR (μg/g)	*n*	SBP	DBP	Pulse pressure
Arsenic		0.08		1.68		0.06		639		0.93 (0.25, 1.62)**		0.17 (–0.20, 0.55)		0.76 (0.22, 1.30)**
Arsenic*c*								466		1.43 (0.34, 2.51)*		0.63 (0.05, 1.21)*		0.80 (–0.07, 1.66)
Cadmium		0.02		1.97		0.02		592		0.22 (–0.10, 0.54)		0.12 (–0.05, 0.28)		0.11 (–0.15, 0.36)
Cadmium*c*								495		0.21 (–0.13, 0.56)		0.08 (–0.10, 0.27)		0.13 (–0.15, 0.41)
Mercury		0.22		2.40		0.31		495		–0.56 (–2.27, 1.16)		–0.21 (–1.16, 0.75)		–0.35 (–1.70, 1.00)
Mercury*c*								428		–0.76 (–2.68, 1.16)		–0.27 (–1.32, 0.78)		–0.49 (–2.00, 1.02)
Manganese		0.28		8.82		0.40		639		–0.45 (–1.18, 0.28)		–0.12 (–0.51, 0.28)		–0.33 (–0.91, 0.25)
Manganese*c*								465		–1.09 (–2.08, –0.10)*		–0.62 (–1.15, –0.09)*		–0.47 (–1.26, 0.32)
Lead		0.31		14.70		0.52		639		–0.07 (–0.67, 0.53)		0.24 (–0.08, 0.57)		–0.31 (–0.78, 0.16)
Lead*c*								464		–0.22 (–0.93, 0.50)		0.27 (–0.11, 0.65)		–0.49 (–1.06, 0.08)
**a**For each metal, the same IQR value is examined in relation to BP for both metal-adjusted and non-metal-adjusted models. **b**All regression models are adjusted for age, cigarette smoking, pack-years of smoking, season of clinical visit, year of clinical visit, BMI, years of education, race/ethnicity, and alcohol intake. Mercury-BP models are adjusted for fish intake. **c**In addition to the covariates in footnote *b*, this regression model is also adjusted for the four other toenail metals. *p < 0.05. **p < 0.01.														

*Arsenic.* An IQR (0.06 µg/g) increase in arsenic was associated with higher SBP [0.93 mmHg; 95% confidence interval (CI): 0.25, 1.62] and pulse pressure (0.76 mmHg; 95% CI: 0.22, 1.30) and showed a weak positive association with DBP (Table 2). Adjustment for other metals strengthened associations with SBP (1.43 mmHg, 95% CI: 0.34, 2.51) and DBP (0.63 mmHg, 95% CI: 0.05, 1.21). The association with pulse pressure was essentially unchanged but was less precise (Table 2).

We evaluated the dose–response relationship between arsenic and SBP, using penalized splines [Supplemental Material, Figure 1 (http://dx.doi.org/10.1289/ehp.1002805)]. Out of a choice of up to 10 degrees of freedom, generalized cross validation chose a slope of 1, indicating a linear association. Thus, there is no evidence of deviation from linearity in the arsenic–SBP model.

*Cadmium.* An IQR (0.02 µg/g) increase in cadmium was not significantly associated with BP (Table 2). Adjustment for other metals did not meaningfully alter these results (Table 2). Assigning participants with cadmium levels below the DL a concentration value of DL/2 did not appreciably alter results (data not shown).

*Mercury.* An IQR (0.31 µg/g) increase in mercury was negatively but not significantly associated with BP (SBP: –0.56, 95% CI: –2.27, 1.16; DBP: –0.21 mmHg, 95% CI: –1.16, 0.75; pulse pressure: –0.35 mmHg, 95% CI: –1.70, 1.00) (Table 2). Adjustment for other metals did not meaningfully affect these results (Table 2). Mercury regressions have a considerably smaller sample size than regressions for other metals (Table 2) because of toenail samples of insufficient weight to assess mercury concentrations as well as adjustment for fish and shellfish intake.

*Manganese.* Manganese was negatively but not significantly associated with BP (SBP: –0.45 mmHg, 95% CI: –1.18, 0.28; DBP: –0.12 mmHg, 95% CI: –0.51, 0.28; pulse pressure: –0.33 mmHg, 95% CI: –0.91, 0.25) (Table 2). After adjusting for other metals, an IQR (0.40 µg/g) increase in manganese was more strongly associated with decreased SBP (–1.09 mmHg; 95% CI: –2.08, –0.10) and DBP (–0.62 mmHg; 95% CI: –1.15, –0.09).

*Lead.* An IQR (0.52 µg/g) increase in lead was not associated with BP (SBP: –0.07 mmHg, 95% CI: –0.67, 0.53; DBP: 0.24 mmHg, 95% CI: –0.08, 0.57; pulse pressure: –0.31 mmHg, 95% CI: –0.78, 0.16) (Table 2). Adjustment for other metals did not meaningfully alter these results (Table 2).

*Hypertension.* Estimated associations of hypertension with an IQR increase in toenail metals were consistent with linear regression results [arsenic, odds ratio (OR) = 1.07, 95% CI: 0.97, 1.20; cadmium, OR = 1.01, 95% CI: 0.95, 1.06; mercury, OR = 0.85, 95% CI: 0.68, 1.05; manganese, OR = 0.96, 95% CI: 0.87, 1.05; lead, OR = 1.00, 95% CI: 0.92, 1.08]. Upon adjusting for other metals, manganese was negatively associated with hypertension (OR = 0.88; 95% CI: 0.77, 0.999). Similar adjustment did not meaningfully alter the other results (data not shown).

*Effect modification.* We found a significant interaction between arsenic and statins for DBP but not for SBP or pulse pressure (Table 3). Among men not taking statins (*n* = 404), an IQR increase in arsenic was associated with an increase in DBP (0.76 mmHg; 95% CI: 0.20, 1.33). Arsenic showed a nonsignificant negative association with DBP among statin users (*n* = 235; –0.30 mmHg; 95% CI: –0.80, 0.21). Within the sample included in arsenic–BP analyses, statin and antihypertensive use were strongly associated with each other based on chi-square analysis and age-adjusted logistic regression (data not shown).

**Table 3:** Effect modification of the association between an IQR increase in toenail arsenic level*a* and BP by statin use (*n* = 639).*b*

Change in BP (mmHg) (95% CI)				
Current statin use	SBP	DBP	Pulse pressure
No (*n* = 404)		1.46 (0.45, 2.47)**		0.76 (0.20, 1.33)**		0.69 (–0.08, 1.46)
Yes (*n* = 235)		0.64 (–0.32, 1.61)		–0.30 (–0.80, 0.21)		0.94 (0.15, 1.74)*
*p*-Value for interaction		0.23		0.01		0.81
**a**IQR for arsenic: 0.06 μg/g. **b**Regression models were adjusted for age, cigarette smoking, pack-years of smoking, season of clinical visit, year of clinical visit, BMI, years of education, race/ethnicity, and alcohol intake. *p < 0.05. **p < 0.01.						

We found no significant interactions between manganese and statins (SBP: *p* = 0.76, DBP: *p* = 0.39; pulse pressure: *p* = 0.84). Results were similar after adjustment for other metals (data not shown).

## Discussion

In a cohort of elderly men with low toenail arsenic concentrations, we found positive associations between toenail arsenic and BP levels. Our results also tentatively suggest negative associations between toenail manganese and BP levels. We found little evidence of associations with toenail cadmium, mercury, or lead. If confirmed, these results may be important because of the high prevalence of both cardiovascular disease and low to moderate metal exposures in the United States.

No investigations have assessed relations between arsenic, cadmium, mercury, manganese, or lead and BP using toenails as biomarkers. Toenail samples are convenient to collect and store, grow more slowly than hair, are more protected from external contaminants, and represent longer-term exposures than blood or urine (Slotnick and Nriagu 2006). Toenails likely reflect metal exposures from the preceding 12–18 months, with slower growth among the elderly (Slotnick and Nriagu 2006).

*Arsenic.* Arsenic may elevate BP through pathways related to oxidative stress (Valko et al. 2005), inflammation (Wu et al. 2003), and endothelial dysfunction/nitric oxide inhibition (Pi et al. 2000).

Epidemiologic studies have mostly evaluated inorganic arsenic, which is more toxic than organic arsenic. Our toenail assay does not distinguish between forms. However, seafood is the main source of organic arsenic (Slotnick and Nriagu 2006). Toenail arsenic is very weakly or not at all correlated with fish (dark-meat fish: *r* = 0.06; other fish: *r* = 0.09; tuna: *r* = -0.004) and shellfish (*r* = 0.06) intake among our participants.

Arsenic sources include soil, dust, air, and food (Benbrahim-Tallaa and Waalkes 2008; Slotnick and Nriagu 2006). Water is a potentially important inorganic arsenic source (Slotnick and Nriagu 2006). The Massachusetts Water Resources Authority (MWRA), in which arsenic is consistently undetectable (< 1.0 µg/L) (MWRA 2011), supplies most of the Greater Boston area.

Arsenic is found in toenails because of its high affinity to sulfhydryl groups (Karagas et al. 2000). Toenails are validated biomarkers of arsenic exposure (Slotnick et al. 2007; Slotnick and Nriagu 2006). Toenail concentrations in our study appear similar to those reported in most U.S. general population studies (Garland et al. 1993; Karagas et al. 2001; Kwong et al. 2010; Slotnick et al. 2005, 2007; Tsuji et al. 2005). Other U.S. studies report somewhat higher (Adair et al. 2006; MacIntosh et al. 1997) or lower (Beane Freeman et al. 2004) arsenic levels.

Several investigations have assessed relations between high-level arsenic exposure and BP. These include two population-based analyses that found dose–response relationships between well-water arsenic and hypertension (Chen et al. 1995; Rahman et al. 1999). Exposure categories used in these studies (10–700, 710–900, > 900 µg/L, and < 500, 500–1,000, > 1,000 µg/L, respectively) are much greater than the maximum contaminant level for arsenic in the United States (10 µg/L), although in practice this target value is often exceeded in U.S. drinking water sources [Agency for Toxic Substances and Disease Registry (ATSDR) 2007]. A recent Iranian study also found positive associations between high-level arsenic exposure and BP (Dastgiri et al. 2010).

Two population-based studies in Bangladesh and Inner Mongolia (with exposure categories of < 8, 8.1–40.8, 40.9–91.0, 91.1–176.0, 176.1–864.0 µg/L, and < 20, 21–50, 51–100, > 100 µg/L drinking water, respectively) reported positive associations between more moderate arsenic levels and BP-related outcomes (Chen et al. 2007; Kwok et al. 2007). A U.S. study found an association between well-water arsenic levels of ≥ 10 µg/L versus < 2 µg/L and self-reported high BP (Zierold et al. 2004).

We found positive associations between arsenic and BP levels, consistent with the current literature. Our study likely evaluated considerably lower arsenic exposure levels than previous investigations, because arsenic is consistently undetectable in the main water supply for the Greater Boston area (MWRA 2011). Our study was the first to use a biomarker of arsenic dose when assessing relations with BP.

*Cadmium.* Cadmium may act on BP through mechanisms related to oxidative stress (Valko et al. 2005), inflammation (Kayama et al. 1995), endothelial dysfunction, partial agonism of calcium channels, increased vasoconstriction, and activation of the sympathetic nervous system (Varoni et al. 2003). It may also act through renal tubular damage, sodium retention, and volume overload (Satarug et al. 2006).

Food from cadmium-contaminated soils is a major exposure source (Satarug and Moore 2004). Other sources include tobacco smoke, soil, dust, water, and air pollution (Henson and Chedrese 2004; Satarug and Moore 2004).

Toenail cadmium concentrations were lower in our study than in other U.S. general population studies (Platz et al. 2002; Slotnick et al. 2005; Yoshizawa et al. 2002). Data on validity of toenails as cadmium biomarkers are sparse. Most relevant studies found associations between cadmium exposure and concentrations in toenails or nails (Anwar 2005; Mortada et al. 2002; Vinceti et al. 2007). However, toenail levels did not vary with smoking (a major cadmium source) among our participants. This may reflect shortcomings of toenails as cadmium biomarkers or other factors, such as the potential influence of nontobacco cadmium sources. In addition, few participants were active smokers, and number of pack-years likely reflects an exposure window too distant to influence current toenail concentrations.

The literature regarding cadmium and BP is inconsistent. Investigations report positive (Satarug et al. 2005; Tellez-Plaza et al. 2008) and null (Staessen et al. 1991, 2000) associations with BP. One ecologic analysis reported a negative relation (Kagamimori et al. 1986). We found little evidence of associations between cadmium and BP. This should be interpreted cautiously in light of an unvalidated biomarker of exposure.

*Mercury.* Mercury may act on BP through mechanisms related to oxidative stress, vascular inflammation, endothelial dysfunction/nitric oxide inhibition, renal tubular dysfunction, and proteinuria (Houston 2007).

Mercury exposure occurs primarily through seafood and dental amalgams (Brodkin et al. 2007). Toenail mercury is a reliable, well-validated biologic marker of long-term exposure to the metal (Garland et al. 1993; Joshi et al. 2003; MacIntosh et al. 1997; Rees et al. 2007). Toenail mercury concentration in our analysis was similar to other general population studies (Garland et al. 1993; Rees et al. 2007) but lower than in the U.S. Health Professionals Follow-Up Study (Joshi et al. 2003).

The overall evidence on mercury and BP is inconsistent, with studies reporting positive and null associations (Johansson et al. 2002; Pedersen et al. 2005; Valera et al. 2009). Data on associations between low-level mercury and BP are sparse. We found little evidence of an association between mercury and BP.

*Manganese.* Manganese may act on BP by lowering vascular sensitivity to alpha-adrenergic receptor activation, decreasing dopamine levels, and inducing oxidative stress. It may also act through antagonism of the alpha-adrenergic receptor in blood vessels, calcium channel antagonism, or effects on the autonomic nervous system (Jiang and Zheng 2005).

Manganese exposure occurs through food, water, air, and soil (Santamaria 2008). Manganese is associated with hypotension and decreased BP in animal (Jiang and Zheng 2005) and occupational research (Jiang et al. 2002; Su et al. 1998). Postural hypotension was observed in manganese dipyridoxyldiphosphate overdosed patients (Misselwitz et al. 1995). Toenail manganese concentration in our investigation is similar, although slightly lower, relative to another U.S. general population study (Slotnick et al. 2005).

Environmental manganese is generally considered to lower hypertension risk (Houtman 1996). However, nonoccupational studies have reported positive (Taneja and Mandal 2007), null (Tubek and Tubek 2008), and negative (Gonzalez-Muñoz et al. 2010) associations with BP-related outcomes. Results may differ across investigations because of variations in study design, confounder control, metal measurement, definition or measurement of the BP outcome, manganese exposure level, and participant characteristics.

We found negative relations between toenail manganese and BP in models adjusted for other metals. This is consistent with results observed in occupational investigations. However, these tentative results should be interpreted extremely cautiously, because toenails are not validated biomarkers of environmental manganese exposure.

*Lead.* Proposed mechanisms linking lead to BP elevation include effects on kidney function, oxidative stress, and nitric oxide and guanylate cyclase levels, and changes in the renin-angiotensin system (Navas-Acien et al. 2007).

Lead is associated with hypertension and BP elevation in occupational and experimental animal research and in many environmental epidemiologic studies (Navas-Acien et al. 2007). A recent systematic review concluded that this association is causal (Navas-Acien et al. 2007). Bone lead, which represents cumulative lead exposure (Hu et al. 2007), is consistently associated with BP outcomes in the NAS (Cheng et al. 2001; Hu et al. 1996; Perlstein et al. 2007). Toenail lead levels in our analysis were similar to those of adults in another U.S. general population study (Slotnick et al. 2005).

Exposure occurs primarily through inhalation of lead dust and ingestion of contaminated food and water (Rosin 2009). Toenails are not validated biomarkers of lead exposure. In fact, some research suggests that nails may be inappropriate biomarkers for lead (Barbosa et al. 2005). We found no association between toenail lead and BP, perhaps because of a number of factors. For example, toenail lead represents a shorter averaging time than bone lead. Alternatively, toenails may be a poor lead biomarker. Among men included in lead–BP analyses, toenail lead was weakly correlated with blood lead (*r* = 0.28, *p* < 0.0001), but not tibia (*r* = 0.04, *p* = 0.55) or patella (*r* = 0.03, *p* = 0.64) lead (blood and bone lead were measured as described by Hu et al. 1996).

*Effect modification.* Statins reduce inflammation and oxidative stress, including in the vascular endothelium, and increase nitric oxide levels (Rubba 2007).

We found statistically significant interaction between arsenic and statins for DBP, with associations limited to statin nonusers. Thus, it is possible that statins blunt effects of arsenic on DBP, although this interaction needs to be evaluated in other studies. We found no evidence of interactions between manganese and statin use.

## Study Limitations and Future Research Directions

The cross-sectional design of our investigation precludes us from ascertaining causality. We had limited power to assess interactions. In addition, our study population consists almost entirely of white, elderly men.

Another limitation is using toenails as biomarkers. Although toenails are validated biomarkers for mercury and arsenic, their validity is unclear for cadmium and manganese and has been called into question for lead. It is very important to interpret results with caution. BP readings are based on only one study visit, although BP is variable and affected by many stimuli. This variability may attenuate results.

We did not correct for multiple comparisons. We have examined five metals. However, they all have pathway-based support for associations with BP, and we believe that our study should not be penalized relative to investigations examining only individual metals under these circumstances.

More research is needed to address relations between metals and BP among larger, more diverse prospective cohorts. Potential interactions between arsenic and statins should be evaluated in future analyses.

## Conclusions

Our findings suggest that environmental arsenic may be associated with BP. Our analyses also tentatively suggest that toenail manganese may be associated with decreased BP. We found no consistent associations with IQR increases in toenail mercury, cadmium, or lead. These findings should be interpreted in light of the difficulty of ascertaining fish intake [the beneficial cardiovascular effects of this important mercury source (Appel et al. 1993) may confound associations between toenail mercury and BP] and potential shortcomings of toenails as biomarkers for metal exposure.

Although changes in BP reported here are not clinically relevant on an individual level, shifting the population distribution of BP has public health significance (Dickinson et al. 2007). Findings of associations for arsenic at these levels, if confirmed, suggests that current efforts to regulate arsenic exposure in the general population may be inadequate.

## Supplemental Material

(700 KB) PDF
