# Supplementary material for: Associations of Toenail Arsenic, Cadmium, Mercury, Manganese, and Lead with Blood Pressure in the Normative Aging Study
Source: Environ Health Perspect. 2011 Aug 30;120(1):98–104. doi: 10.1289/ehp.1002805 (PMC3261928; doi:10.1289/ehp.1002805)

Supplemental Material

Associations of Toenail Arsenic, Cadmium, Mercury, Manganese and Lead with Blood Pressure in the Normative Aging Study

Irina Mordukhovich[1], Robert O Wright[2,3], Howard Hu[4], Chitra Amarasiriwardena[3], Andrea Baccarelli[2,5,] Augusto Litonjua[3], David Sparrow[6], Pantel Vokonas[6], Joel Schwartz[2,3]

[1]Department of Epidemiology, Gillings School of Global Public Health, University of North Carolina, Chapel Hill, North Carolina, USA

[2]Department of Environmental Health, Harvard School of Public Health, Boston, Massachusetts, USA

[3]Channing Laboratory, Department of Medicine, Brigham and Women's Hospital, Harvard Medical School, Boston, Massachusetts, USA

[4]Department of Environmental Health Sciences, School of Public Health, University of Michigan, Ann Arbor, Michigan, USA

[5]Department of Environmental and Occupational Health, IRCCS Maggiore Hospital, Mangiagalli and Regina Elena Foundation and Università degli Studi di Milano, Milan, Italy

[6]VA Normative Aging Study, Veterans Affairs Boston Healthcare System and the Department of Medicine, Boston University School of Medicine, Boston, Massachusetts, USA

Correspondence: Irina Mordukhovich, Department of Epidemiology, CB#7435, McGavran-Greenberg Hall, University of North Carolina, Chapel Hill, NC 27599-7435.

Phone: (919)619-9285. Fax: (919)966-2089. Email: irinam@email.unc.edu

Work for this manuscript was performed at the Harvard School of Public Health, 677 Huntington Avenue, Boston, MA 02115

Supplemental Table 1. Toenail metal concentrations by participant characteristics, reported as median (interquartile range)[a].

Characteristics	Arsenic	Cadmium	Mercury	Manganese	Lead
Age (years)					
<72	0.08 (0.06)	0.01 (0.02)	0.23 (0.37)	0.30 (0.41)	0.28 (0.47)
72+	0.07 (0.06)	0.02 (0.03)	0.19 (0.26)	0.27 (0.40)	0.32 (0.60)
BMI (kg/m^2)					
< 25	0.08 (0.06)	0.02 (0.03)	0.22 (0.25)	0.29 (0.47)	0.34 (0.57)
25 – 29	0.08 (0.07)	0.01 (0.02)	0.22 (0.30)	0.28 (0.39)	0.30 (0.52)
30 +	0.07 (0.05)	0.02 (0.03)	0.18 (0.28)	0.26 (0.50)	0.30 (0.47)
Current smoking status					
Never	0.08 (0.06)	0.02 (0.02)	0.20 (0.28)	0.25 (0.41)	0.30 (0.57)
Former	0.08 (0.06)	0.02 (0.03)	0.23 (0.31)	0.29 (0.40)	0.32 (0.48)
Current	0.09 (0.11)	0.02 (0.02)	0.14 (0.15)	0.38 (0.41)	0.30 (0.64)
Smoking (pack years)					
<30	0.07 (0.07)	0.02 (0.03)	0.22 (0.28)	0.29 (0.40)	0.33 (0.61)
30+	0.08 (0.06)	0.02 (0.02)	0.22 (0.38)	0.29 (0.40)	0.26 (0.41)
Alcohol intake					
< 2 drinks/day	0.07 (0.06)	0.01 (0.02)	0.20 (0.29)	0.28 (0.40)	0.30 (0.49)
2+ drinks /day	0.09 (0.09)	0.02 (0.03)	0.27 (0.41)	0.32 (0.47)	0.38 (0.58)
Season of clinical visit					
Spring	0.07 (0.06)	0.01 (0.03)	0.21 (0.31)	0.24 (0.35)	0.27 (0.69)
Summer	0.09 (0.08)	0.02 (0.03)	0.21 (0.20)	0.35 (0.56)	0.30 (0.46)
Fall	0.08 (0.06)	0.02 (0.02)	0.23 (0.34)	0.30 (0.45)	0.35 (0.58)
Winter	0.07 (0.04)	0.01 (0.02)	0.22 (0.28)	0.24 (0.25)	0.30 (0.46)
Years of education					
< 15	0.08 (0.06)	0.02 (0.03)	0.19 (0.26)	0.30 (0.47)	0.35 (0.53)
15 +	0.08 (0.06)	0.01 (0.02)	0.25 (0.36)	0.26 (0.37)	0.26 (0.47)
Race/ethnicity					
Non-Hispanic White	0.08 (0.06)	0.02 (0.03)	0.21 (0.30)	0.28 (0.40)	0.31 (0.53)
Non-Hispanic Black	0.09 (0.05)	0.01 (0.02)	0.19 (0.14)	0.18 (0.34)	0.28 (0.31)
Antihypertensive use					
No	0.08 (0.07)	0.02 (0.03)	0.23 (0.30)	0.30 (0.45)	0.33 (0.70)
Yes	0.07 (0.06)	0.01 (0.02)	0.19 (0.29)	0.26 (0.39)	0.28 (0.47)
Statin use					
No	0.08 (0.07)	0.02 (0.03)	0.19 (0.29)	0.31 (0.46)	0.33 (0.58)
Yes	0.07 (0.06)	0.01 (0.02)	0.24 (0.35)	0.24 (0.36)	0.24 (0.40)
Year of clinical visit					
1999-2004	0.08 (0.06)	0.02 (0.03)	0.22 (0.31)	0.27 (0.40)	0.32 (0.53)
2005-2009	0.08 (0.06)	0.01 (0.01)	0.16 (0.20)	0.34 (0.44)	0.17 (0.33)
Dark-meat fish intake					
<1 serving/month	0.07 (0.06)	0.02 (0.03)	0.15 (0.19)	0.28 (0.44)	0.37 (0.59)
1/ month – 6/day	0.08 (0.07)	0.02 (0.03)	0.31 (0.39)	0.26 (0.39)	0.30 (0.50)
Other fish intake					
< 1 serving/month	0.07 (0.07)	0.02 (0.03)	0.15 (0.19)	0.27 (0.47)	0.42 (0.74)
1/month – 6/week	0.08 (0.06)	0.02 (0.03)	0.26 (0.35)	0.27 (0.40)	0.30 (0.47)
Shellfish intake					
<1 serving/month	0.07 (0.06)	0.02 (0.03)	0.18 (0.23)	0.27 (0.39)	0.32 (0.59)
1/month – 6/week	0.08 (0.07)	0.02 (0.03)	0.27 (0.36)	0.28 (0.42)	0.32 (0.53)
Intake of canned tuna					
< 1 serving/month	0.07 (0.07)	0.01 (0.03)	0.12 (0.25)	0.28 (0.43)	0.32 (0.56)
1/month – 1/day	0.08 (0.06)	0.02 (0.03)	0.25 (0.33)	0.27 (0.41)	0.33 (0.56)

a. Data is from the first study visit in which participants donated toenail samples and had complete information on blood pressure and covariates (age, cigarette smoking, pack years of smoking, season of clinical visit, year of clinical visit, body mass index, years of education, race, and alcohol intake).

Supplementary Table 2. Correlations between toenail metals, using Spearman's *rho*.

Toenail Metal	Arsenic	Cadmium	Mercury	Manganese	Lead
Arsenic	1.0	0.39#	0.09*	0.56#	0.45#
Cadmium	0.39#	1.0	0.01	0.49#	0.58#
Mercury	0.09*	0.01	1.0	-0.07	-0.01
Manganese	0.56#	0.49#	-0.07	1.0	0.50#
Lead	0.45#	0.58#	-0.01	0.50#	1.0

* $p < 0.05$, ** $p < 0.01$, # $p < 0.0001$

Supplemental Figure 1. Dose-response relationship between toenail arsenic and systolic blood pressure, using a penalized spline. Circles represent individual observations. The shaded area represents the pointwise 95% confidence intervals.

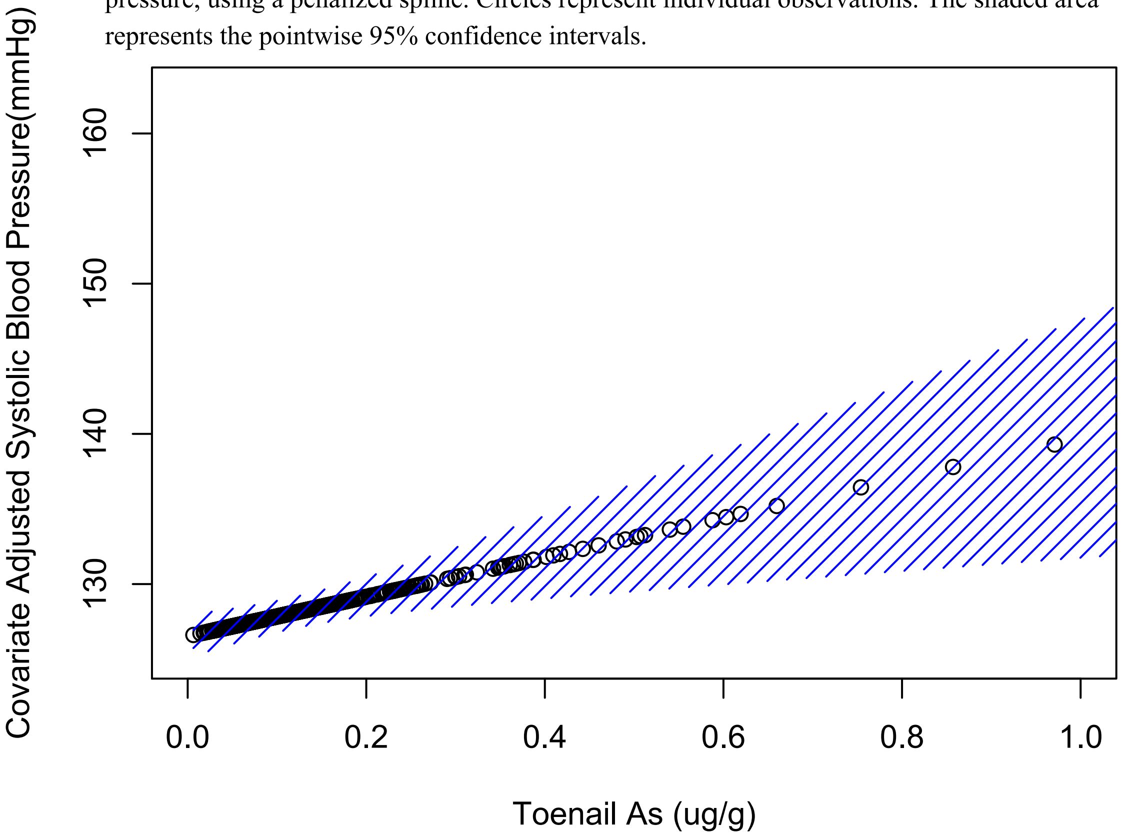

Supplement: (700 KB) PDF [file ehp.1002805.s001.pdf]
